# Reconfigurable Angular Resolution Design Method in a Separate-Axis Lissajous Scanning MEMS LiDAR System

**DOI:** 10.3390/mi13030353

**Published:** 2022-02-23

**Authors:** Fahu Xu, Dayong Qiao, Changfeng Xia, Xiumin Song, Wenhui Zheng, Yaojun He, Qiaodan Fan

**Affiliations:** 1 Key Laboratory of Micro/Nano Systems for Aerospace, Ministry of Education, Northwestern Polytechnical University, Xi’an 710072, China; fahu.xu@mail.nwpu.edu.cn; 2 Shaanxi Province Key Laboratory of Micro and Nano Electro-Mechanical Systems, Northwestern Polytechnical University, Xi’an 710072, China; 3 Ningbo Institute of Northwestern Polytechnical University, Ningbo 315103, China; 4 Xi’An Zhisensor Technologies Co., Ltd., Xi’an 710072, China; xchangfeng@mail.nwpu.edu.cn (C.X.); xiumin.song@zhisensor.com (X.S.); wenhui.zheng@zhisensor.com (W.Z.); yaojun.he@zhisensor.com (Y.H.); qiaodan.fan@zhisensor.com (Q.F.)

**Keywords:** MEMS LiDAR, reconfigurable angular resolution, separate-axis Lissajous scanning

## Abstract

MEMS-based LiDAR with a low cost and small volume is a promising solution for 3D measurement. In this paper, a reconfigurable angular resolution design method is proposed in a separate-axis Lissajous scanning MEMS LiDAR system. This design method reveals the influence factors on the angular resolution, including the characteristics of the MEMS mirrors, the laser duty cycle and pulse width, the processing time of the echo signal, the control precision of the MEMS mirror, and the laser divergence angle. A simulation was carried out to show which conditions are required to obtain different angular resolutions. The experimental results of the 0.2° × 0.62° and 0.2° × 0.15° (horizontal × vertical) angular resolutions demonstrate the feasibility of the design method to realize a reconfigurable angular resolution in a separate-axis Lissajous scanning MEMS LiDAR system by employing MEMS mirrors with different characteristics. This study provides a reasonable potential to obtain a high and flexible angular resolution for MEMS LiDAR.

## 1. Introduction

In recent years, light detection and ranging (LiDAR) as a 3D optical imaging technology has been explored extensively and has received much attention in the field of autonomous driving, robots, and automatic guided vehicles (AGVs) [1,2,3,4,5]. With the advancement of self-driving technologies, 3D object recognition and tracking increase the demand for high-angular-resolution LiDAR systems. Nevertheless, for the conventional mechanical rotary LiDAR systems with generally a low vertical resolution [6], a higher angular resolution means that more transmitting and receiving elements are required, which leads to a larger volume and higher cost. MEMS-based LiDAR employing a small-size and fast-speed MEMS mirror as the scanner shows a unique advantage in achieving a high and flexible angular resolution [7,8,9,10].

In a MEMS-based LiDAR system, the MEMS mirror as the crucial component is employed to realize 1D or 2D scanning. With the real-time movement of the MEMS mirror, the angular resolution can be achieved by emitting laser pulses at the predefined mirror positions. To obtain a high angular resolution in a MEMS-based LiDAR system, a MEMS mirror with a large field of view (FOV), high frequency, and large size is needed. However, there exists a design trade-off between the scanning speed, size, and tilt angle of the MEMS mirror [11,12,13]. A two-axis MEMS mirror usually suffers from a small optical scanning angle, which limits the angular resolution in terms of optics. Additionally, the cross-talk between the two orthogonal axes of the two-axis MEMS mirror also restricts the angular resolution in terms of the control precision. A uniaxial MEMS mirror with a relatively simple design and fabrication shows a promising potential to obtain a large FOV and a high frequency simultaneously. For instance, Gu-Stoppel et al. presented a single-axial resonant-driven microscanner with an optical FOV of 73.2° and a high frequency of 27 kHz [14]. Schwarz et al. demonstrated a resonant 1D MEMS mirror achieving mechanical scanning angles exceeding ±45° [15]. Without the cross-talk problem, more stable and precise feedback control of the uniaxial MEMS mirror can be realized.

Based on the above, a combination of two uniaxial MEMS mirrors is more readily able to obtain a high angular resolution in a MEMS LiDAR system. In our earlier study, we proposed and set up a semi-coaxial MEMS-based LiDAR system that integrates three uniaxial MEMS mirrors [16]. In this paper, we specifically reveal a reconfigurable design method on the angular resolution in a separate-axis Lissajous scanning MEMS LiDAR system, in which two uniaxial MEMS mirrors are employed. Based on this design method, the separate-axis Lissajous scanning MEMS LiDAR system can employ larger-amplitude, higher-frequency MEMS mirrors to obtain a higher angular resolution. A detailed analysis of influence factors on the angular resolution is revealed in this design method, including the characteristics of the MEMS mirrors, the laser duty cycle and pulse width, the processing time of the echo, the control precision of the MEMS mirror, and the laser divergence angle. This design method also describes the procedure of realizing different angular resolutions in a Lissajous scanning MEMS LiDAR system in detail. A simulation was carried out to demonstrate the requirements of realizing a reconfigurable angular resolution. The experiment results of the 0.2° × 0.62° and 0.2° × 0.15° (horizontal × vertical) angular resolutions prove the feasibility of the design method in achieving a reconfigurable angular resolution in a separate-axis Lissajous scanning MEMS LiDAR system. This reconfigurable angular resolution design method demonstrates a new research direction to achieve a high and flexible LiDAR angular resolution.

The rest of this paper is organized as follows: Section 2 presents the analysis of the influence factors on the angular resolution in a Lissajous scanning MEMS LiDAR system. Section 3 shows the design method and simulation to obtain a reconfigurable angular resolution. In Section 4, the experiments are carried out to verify the feasibility of the reconfigurable angular resolution design method in a separate-axis Lissajous scanning MEMS-based LiDAR system. In Section 5, the conclusion is presented.

## 2. Angular Resolution in a Separate-Axis Lissajous Scanning MEMS LiDAR System

In a MEMS-based LiDAR system, a biaxial MEMS mirror with a high frequency, a large scanning angle, and relatively large size is required to achieve a high angular resolution. However, there exists a design trade-off between the scanning speed, size, and tilt angle of the MEMS mirror [15]. On this basis, a separate-axis Lissajous scanning design consisting of two independent uniaxial MEMS mirrors provides a new solution to achieve a reconfigurable and high angular resolution. The concept of this design has been proposed in our earlier research [16,17]. In this study, further investigation on a reconfigurable design method of achieving different angular resolutions was conducted, as shown below.

**Figure 1:**
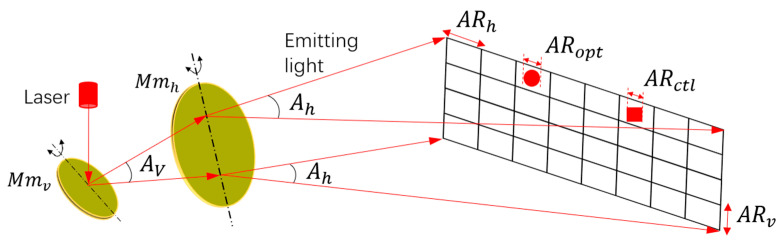
Angular resolution in a separate-axis Lissajous scanning MEMS LiDAR system.

Typically, the angular resolution of a MEMS-based LiDAR system depends on two aspects. One aspect is the control angular resolution of the MEMS mirror ($AR_{ctl}$), which is related to the driving [17,18,19] and feedback control [20,21,22] of the MEMS mirror itself. In the current MEMS-based LiDAR system, the $AR_{ctl}$ is usually controlled under 0.2° in terms of the optical scanning amplitude of the MEMS mirror. The other aspect is the optical resolution ($AR_{opt}$), which mainly depends on the divergence angle of the laser. Due to the millimeter scale of the MEMS mirror, a complicated collimation lens design is generally required to suppress the laser divergence angle [23,24,25]. In order to demonstrate the angular resolution in the separate-axis Lissajous scanning MEMS-based LiDAR system in detail, Figure 1 is depicted below. Here, $Mm_{v}$ represents the vertical scanning MEMS mirror, and $Mm_{h}$ represents the horizontal scanning MEMS mirror. $A_{v}$ means the vertical optical scanning angle, and $A_{h}$ is the horizontal optical scanning angle. In Figure 1, $AR_{h}$ is the horizontal angular resolution, and $AR_{v}$ is the vertical angular resolution. $AR_{opt}$ represents the optical angular resolution, and $AR_{ctl}$ represents the control angular resolution.

Actually, the $AR_{ctl}$ and $AR_{opt}$ define the upper limit of the angular resolution. The final performance of the angular resolution in a Lissajous scanning MEMS LiDAR system depends on more factors. First of all, the characteristics of the MEMS mirror mainly determine the final angular resolution to display in a Lissajous scanning system, including its vibration frequency, scanning angle, and size. Since the angular resolution can be defined by dividing the scanning angle by the pixel number, Formulas (1) and (2) show the theoretical calculation of the $AR_{h}$ and $AR_{v}$ in the Lissajous scanning MEMS LiDAR system [26,27,28,29,30]. Here, $F_{r}$ represents the scanning frame rate which is usually above 15Hz for high-speed vehicle navigation to meet the needs of real-time detection [31]. $F_{v}$ means the vibration frequency of the $Mm_{v}$, $D_{v}$ is the aperture of the $Mm_{v}$, k is a constant which can be considered as 1.27, and λ is the laser wavelength. As can be seen, a higher $F_{v}$ and a larger $D_{v}$, $A_{v}$, and $A_{h}$ are simultaneously needed to achieve a higher angular resolution. Thus, the separate-axis Lissajous scanning design provides a reconfigurable approach to obtain a high and flexible angular resolution, as shown in Figure 1.

(1) $$AR_{h}\geq \frac{\pi F_{r}A_{h}}{2F_{v}}$$

(2) $$AR_{v}\geq \frac{k\lambda A_{v}}{D_{v}}$$

The second factor is that the laser itself has a duty cycle, which represents the time for the laser to be triggered. Theoretically, as long as Formulas (1) and (2) are satisfied, a fairly high angular resolution can be obtained. However, the laser needs response time to store or release energy, which means the times of the laser emission in one second are restricted. As a result, the angular resolution at a certain frame rate is limited by the laser duty cycle. The following formulas show the relationship between the angular resolution and the laser duty cycle, in which $N_{pixel}$ means the number of ranged pixels in one second, $DC_{laser}$ represents the laser duty cycle, and $PW_{laser}$ means the laser pulse width. Formula (3) shows how to calculate the number of ranged pixels in one second. Formula (4) reveals that $N_{pixel}$ cannot exceed the number of laser emissions. Otherwise, the laser could be damaged to some extent. As can be seen, the laser duty cycle also has an impact on the angular resolution. Additionally, a narrower laser pulse width can bring a larger number of laser emissions and then a higher angular resolution.

(3) $$N_{pixel}=\frac{A_{v}}{AR_{V}}\times \frac{A_{h}}{AR_{h}}\times F_{r}$$

(4) $$N_{pixel}\leq \frac{DC_{laser}}{PW_{laser}}$$

Another factor is the processing time of the echo signal ($T_{p}$), including the acquisition time of echo data and the execution time of the ranging algorithm. In the Lissajous scanning system, the scanning speed at different pixel positions varies with time, and the time interval between the current pixel and the next unrepeatable pixel ($T_{i}$) is not a constant. To obtain the ranging information of each pixel in real time, the processing time of the echo signal is preferred to be shorter than the minimum $T_{i}$. Formula (5) shows the ideal relationship between the angular resolution, $T_{i}$, and $T_{p}$ in this separate-axis Lissajous scanning system. Due to the higher vibration frequency of the vertical scanning MEMS mirror, the minimum $T_{i}$ is mainly decided by the $AR_{v}$. As can be seen, a higher angular resolution requires a shorter $T_{p}$ than $T_{i}$. Formula (6) shows this situation regarding the $F_{r}$, $T_{i}$, and $T_{p}$, in which *N* is the total number of pixels in one frame rate accorded with the angular resolution. $F_{r}$ is also limited by the $T_{p}$, and its maximum value is determined by the greatest common divisor (GCD) of the $F_{v}$ and $F_{h}$. As a result, the processing time of the echo signal $T_{p}$ influences the angular resolution. In the $T_{p}$, the acquisition time of echo data is usually a constant value, which can be obtained by dividing the double detected distance by the velocity of light. The execution time of the ranging algorithm is mainly decided by its complexity, and this time can be optimized. As can be seen, a shorter $T_{p}$ means that a higher angular resolution at a certain frame rate can be achieved.

(5) $$T_{i}\geq \frac{\arcsin\left(\frac{AR_{v}}{A_{v}}\right)}{2\pi F_{v}}\geq T_{p}$$

(6) $$F_{r}=\frac{1}{\sum_{i=1}^{N}\max\left(T_{i},T_{p}\right)}\leq GCD\;of\;F_{v}\;\mathrm{and}\;F_{h}$$

As mentioned above, multiple factors influence the real performance of the LiDAR angular resolution. The $AR_{ctl}$ and $AR_{opt}$ define the upper limit of the angular resolution. According to Formula (1) to Formula (6), a high $F_{v}$, a large $D_{v}$, $A_{v}$, and $A_{h}$, a narrow $PW_{laser}$, and a short $T_{p}$ are simultaneously needed to achieve a high angular resolution. Thanks to the independence of the $Mm_{v}$ and $Mm_{h}$, a reconfigurable and high angular resolution can be realized in a separate-axis Lissajous scanning MEMS LiDAR system by employing different characteristic uniaxial MEMS mirrors.

## 3. Design Method and Simulation on Angular Resolution

**Figure 2:**
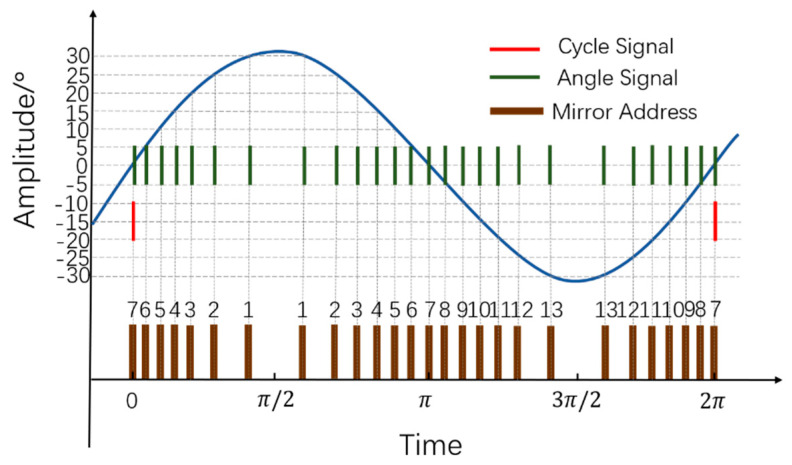
Position signals of the uniaxial MEMS mirror (angular resolution is 5° and optical scanning angle is 60°).

The design method to obtain a reconfigurable angular resolution in a separate-axis Lissajous scanning LiDAR system is presented below. In the MEMS-based LiDAR system, the achievement of the angular resolution depends on the procedure that the laser pulse exactly strikes at the predefined vibration position of the MEMS mirror. Then, it becomes critical to acquire the real-time vibration position of the MEMS mirror and emit the laser pulse accurately. Generally, the MEMS mirror module integrating the driving and feedback control system provides the real-time position information of the MEMS mirror. Figure 2 depicts the situation of the real-time position signals of the uniaxial MEMS mirror. As an example, the angular resolution is 5° and the optical scanning angle is 60° in Figure 2. It should be noted that under a different angular resolution and optical scanning angle, the position signal changes accordingly. As shown, the angle signal, which accords with the angular resolution, represents the real-time angle position of the MEMS mirror. The cycle signal represents the original position in a scanning period of the uniaxial MEMS mirror. In this condition, one scanning cycle of the MEMS mirror starts with the cycle signal, and the angular resolution can be achieved by timely emitting laser pulses to strike on the MEMS mirror with the coming of the angle signals.

To achieve 2D scanning, the cycle signal and angle signals of the $Mm_{v}$ and the $Mm_{h}$ are all needed to confirm the position of each pixel. To obtain an accurate angular resolution, the angle signals of the $Mm_{v}$ and the $Mm_{h}$ are encoded as the vertical scanning mirror moving address ($Addr_{v}$) and the horizontal scanning mirror moving address ($Addr_{h}$), as shown in Figure 2. In this way, the position of each pixel is only defined by the mirror angular address consisting of the $Addr_{v}$ and $Addr_{h}$. Additionally, the laser pulse can be triggered at different angular addresses to achieve a reconfigurable angular resolution. It should be noted that the encoded mirror moving address is not continuously in one scanning period of the uniaxial MEMS mirror. Due to the harmonic motion, the same trajectory is scanned twice in one scanning period of the uniaxial MEMS mirror. In this case, the cycle signal represents the 0 degree of the optical scanning angle and thus the exact middle position of the scanning trajectory. Thus, the first angle signal is encoded as address 7 in the condition of a 5° angular resolution and a 60° optical scanning angle. Similarly, when the time is π/2 or 3π/2, the encoded address is address 1 or 13, which represents a ±30° optical scanning angle. Then, the vibration position signals of the $Mm_{v}$ and the $Mm_{h}$ are all encoded as the angular addresses accorded with the angular resolution. A reconfigurable angular resolution can be achieved by emitting laser pulses at the different angular addresses.

**Figure 3:**
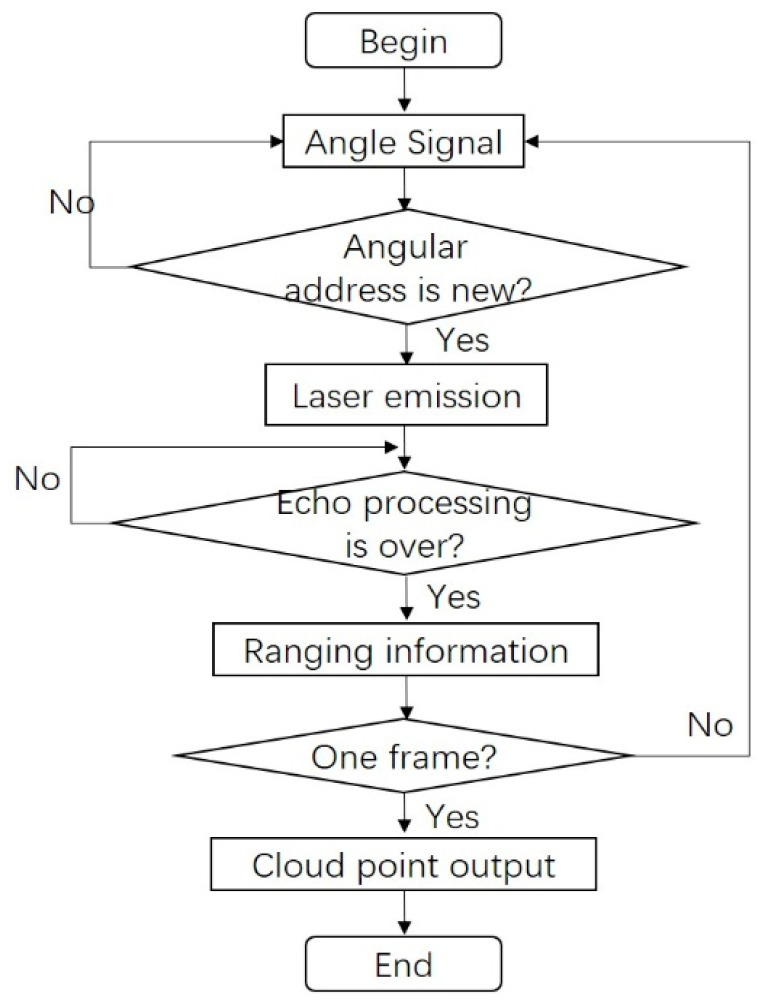
Procedure of reconfigurable angular resolution design method.

In fact, more aspects need to be considered in the procedure of realizing the reconfigurable angular resolution. At first, due to the scanning form of Lissajous, some pixel positions may be scanned repeatedly in one frame rate. In order to utilize the laser duty cycle effectively, the laser pulse should be prevented from hitting those pixels with the same angular address. Secondly, due to the varying scanning speed of Lissajous scanning at different pixel positions, the processing time of the echo signal $T_{p}$ should be considered, and the laser pulse should be triggered after the echo processing is over. Based on the above, the entire realization process of the design method on the angular resolution in the separate-axis Lissajous scanning MEMS LiDAR system is depicted in Figure 3. As shown below, the angle signal of the $Mm_{v}$ or the $Mm_{h}$ can be firstly detected in the ranging procedure. Then, it should be distinguished whether the angular address is repeated or not. If not, the laser pulse can be triggered, and the echo processing unit starts to work. The new angle signal should not be judged until the ranging process is finished. Then, the ranging information of the target can be obtained, including the angular address and detected distance. When all of the unrepeated angular addresses have been detected, the cloud point data of a whole frame can be transmitted to the software to display. Through this design method, reconfigurable angular resolutions can be achieved in the separate-axis Lissajous scanning MEMS LiDAR system.

In order to demonstrate the effect of different factors on the angular resolution of the MEMS LiDAR system, a simulation based on the above design method was conducted, as shown below. As mentioned above, the $AR_{ctl}$, the $AR_{opt}$, the scanning frequency and angle of the $Mm_{v}$ and the $Mm_{h}$, the pulse width and duty cycle of the laser, and the processing time of the echo algorithm all have an impact on the angular resolution. Typically, the $AR_{ctl}$, $AR_{opt}$, $A_{h}$, $A_{v}$, and laser duty cycle are set in advance in a MEMS LiDAR system. In this case, the $AR_{ctl}$ was assumed to be 0.05°, the $AR_{opt}$ was assumed to be 0.1°×0.1°(h×v), $DC_{laser}$ was 0.1%, and $A_{h}\times A_{v}$ was 60°×10°. Based on the assumed parameters and the procedure shown in Figure 3, the simulation on the angular resolution was conducted. Table 1 shows the required parameters of the pulse width of the laser, the frequency of the $Mm_{v}$, and the processing time of the echo to achieve different angular resolutions. As can be seen, the high frequency of the vertical scanning MEMS mirror mainly determines the angular resolution of the MEMS LiDAR system. A narrower width of the laser pulse and a shorter echo processing time are also required to achieve a higher angular resolution. It should be noted that this simulation shows the feasibility of the separate-axis Lissajous scanning MEMS LiDAR system to achieve a reconfigurable angular resolution and provides a promising potential to a high-angular-resolution MEMS LiDAR system.

## 4. Experiments

According to the above analysis and simulation on the reconfigurable angular resolution design method, a separate-axis Lissajous scanning MEMS LiDAR system was set up [16], and the experiments were carried out as described below. Two different angular resolutions were demonstrated in the experiments to reveal the feasibility of the reconfigurable angular resolution design method, including 0.2° × 0.62° (horizontal × vertical) and 0.2° × 0.15° (horizontal × vertical).

In the setup of this separate-axis Lissajous scanning MEMS LiDAR system, an OSRAM pulsed laser diode with a peak power of 75 W and a wavelength of 905 nm was employed as the laser emitter, which can emit narrow laser pulses with a relatively high energy. An Analog Devices high-speed analog-to-digital converter (ADC) with a 500 MHz sampling rate was implemented to digitize the echo signal. The high sampling rate of the ADC determines the ranging accuracy. A time of flight (ToF)-based full-waveform echo processing algorithm was operated and accelerated in the Xilinx Field Programmable Gate Array (FPGA). The parallel processing feature of the FPGA can effectively shorten the echo signal processing time. Then, the distance information of the target is obtained after the echo processing. With the cooperation of the FPGA and microcontroller unit (MCU), the point cloud data were finally generated and transferred to display on the software interface. The entire control procedure of the reconfigurable angular resolution design is shown in Figure 3. It should be noted that the uniaxial MEMS mirrors were provided by Zhisensor Technology [32], including the MEMS mirror module named P1223 employed as the $Mm_{v}$ and the MEMS mirror module named P1220 employed as the $Mm_{h}$. The MEMS mirror modules also provide the driving and feedback control of the MEMS mirror so that precise control of the MEMS mirror can be achieved [16].

As analyzed above, several factors affect the angular resolution. In this separate-axis Lissajous scanning MEMS LiDAR system, the influence factors are shown below. The $AR_{ctl}$ is up to 0.05°, which is determined by the high control precision of the MEMS mirror module. With the optical collimation, the design of the laser divergence angle is optimized to 0.2° × 0.15° (horizontal × vertical) so that the $AR_{opt}$ is defined as 0.2° × 0.15°. The $DC_{laser}$ is 0.1%, which is supported by the laser itself. The optical scanning angle of the $Mm_{v}$ is 10°, and the optical scanning angle of the $Mm_{h}$ is 60°. The vibration frequency of the $Mm_{v}$ is 11,040 Hz, and the vibration frequency of the $Mm_{h}$ is 220 Hz. Thanks to the hardware-accelerated processing of the FPGA, the total execution time of the echo processing $T_{p}$ is controlled in 1.35 us with a maximum detected distance of 20 m. In summary, the specifications of the influence factors of the angular resolution in this separate-axis Lissajous scanning MEMS LiDAR system are illustrated in Table 2.

**Figure 4:**
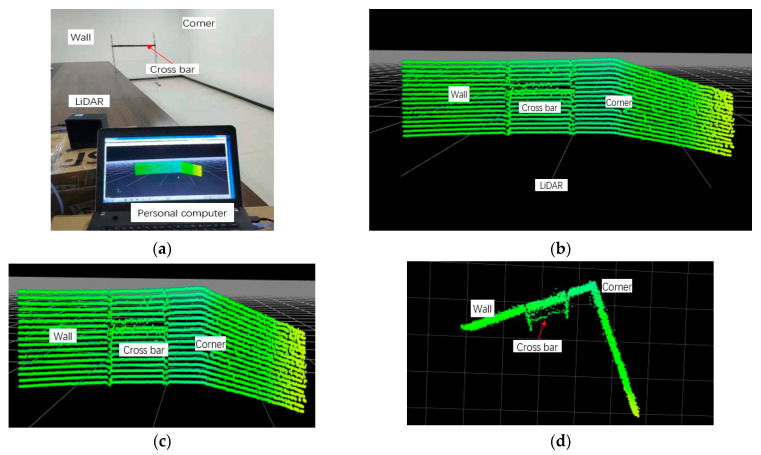
(**a**) Picture of the experiment of the angular resolution of 0.2° × 0.62°, (**b**) panorama of the cloud point, (**c**) front view, and (**d**) top view.

**Figure 5:**
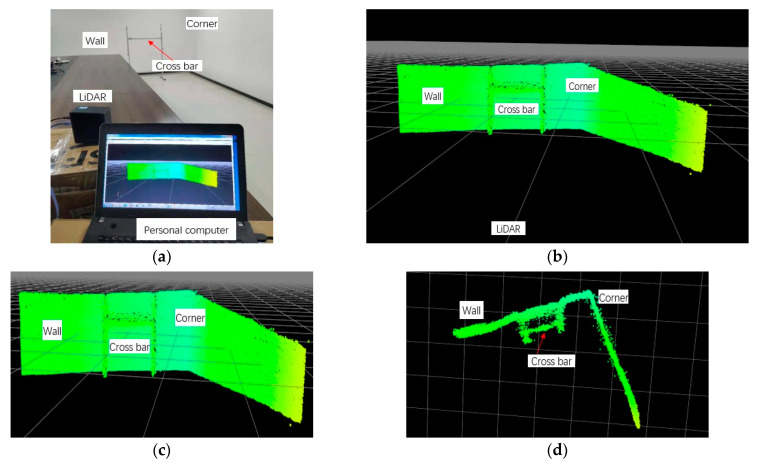
(**a**) Picture of the experiment of the angular resolution of 0.2° × 0.15°, (**b**) panorama of the cloud point, (**c**) front view, and (**d**) top view.

Based on the above parameters provided by the setup of the MEMS LiDAR system, two experiments with different angular resolutions were conducted to demonstrate the feasibility of the reconfigurable design method on the angular resolution in this separate-axis Lissajous scanning MEMS LiDAR system. The entire control procedure of the experiments is shown in Figure 3. Limited by the processing of the hardware circuit, we set up the ranging experiments in a meeting room as shown below. Based on the above analysis, a 0.2° × 0.62° (horizontal × vertical) angular resolution of the separate-axis MEMS LiDAR system with 301 × 17 pixels and a frame rate of 19 Hz was achieved in the first experiment, as shown in Figure 4. In the second experiment, a 0.2° × 0.15° (horizontal × vertical) angular resolution of the separate-axis MEMS LiDAR system with 301 × 65 pixels and a frame rate of 1 Hz was finally obtained, as shown in Figure 5. The above experimental results are consistent with the simulation results in Table 1. As can be seen, two different angular resolutions were obtained in these experiments, which means that a reconfigurable angular resolution can be achieved when MEMS mirrors with different characteristics are employed in the separate-axis MEMS LiDAR system.

In the ranging image of the experiments, a bracket, the background wall, and the corner were ranged as the targets, in which the bracket was placed at approximately 6 m in front of the MEMES LiDAR system, and the background wall was situated at approximately 7 m. It should be noted that the 0.2° horizontal angular resolution has been proven in our earlier study [16]. In experiment 1, the bracket with a 6 cm-wide crossbar was placed in front of the MEMS LiDAR system, and the 0.62° vertical angular resolution with 17 pixels can be seen from the cloud point image, as shown in Figure 4. In experiment 2, the bracket with a 2 cm-wide crossbar was placed in front of the MEMS LiDAR system, and Figure 5 shows the 0.15° vertical angular resolution with 65 pixels in the ranging experiment. However, due to the limitation of the design and processing ability of the hardware circuit, the cloud point noise appears on the object boundary. Nonetheless, these experiments provide support for the reconfigurable design method on the angular resolution in this separate-axis Lissajous scanning MEMS LIDAR system. In our next work, we will focus on improving this design to provide much better 3D ranging images.

## 5. Conclusions

In this paper, we propose a reconfigurable angular resolution design method in a separate-axis Lissajous scanning MEMS LiDAR system. This design method reveals the procedure of achieving different angular resolutions in a Lissajous scanning MEMS LiDAR system and demonstrates the influence of different factors on the angular resolution, including the characteristics of the MEMS mirrors, the laser duty cycle, the laser pulse width, the processing time of the echo, the control precision of the MEMS mirror, and the laser divergence angle. The simulation showed which conditions are required to obtain a high angular resolution, and experiments with different angular resolutions were conducted. The experiment results show the feasibility of the reconfigurable angular resolution design method in a separate-axis Lissajous scanning MEMS LiDAR system. Based on this design method, the separate-axis Lissajous scanning MEMS LiDAR system can employ larger-amplitude, higher-frequency MEMS mirrors to obtain a higher angular resolution. In our next study, more improvements of this design will be researched to provide a promising solution to high-angular-resolution MEMS LiDAR.

## Figures and Tables

**Table 1:** Parameters for different angular resolutions.

Angular Resolution (*h* × *v*)	*Pixels* (*h* × *v*)	*F_v_*	*PW_laser_*	*T_p_*	*F_r_*
1° × 1°	61 × 11	1436 Hz	99 ns	11.08 us	15 Hz
0.5° × 1°	121 × 11	2489 Hz	50 ns	2.79 us	15 Hz
0.2° × 1°	301 × 11	7088 Hz	20 ns	449 ns	15 Hz
0.2° × 0.62°	301 × 17	11,040 Hz	10 ns	1.35 us	19 Hz
0.2° × 0.15°	301 × 65	11,040 Hz	10 ns	1.35 us	1 Hz

**Table 2:** Influence factors on angular resolution.

Characteristic	Value
$AR_{ctl}$	0.05°
$AR_{opt}$	0.2°×0.15° (h×v)
$DC_{laser}$	0.1%
$PW_{laser}$	10 ns
$T_{p}$	1.35 us
$A_{v}\times A_{h}$	10°×60°
$F_{v}\times F_{h}$	11,040 Hz × 220 Hz
